# Effect of Workplace-Based Assessment Utilization as a Formative Assessment for Learning Among Family Medicine Postgraduates at the Faculty of Medicine, Menoufia University: A Prospective Study

**DOI:** 10.7759/cureus.35246

**Published:** 2023-02-21

**Authors:** Safa H Alkalash, Nagwa A Farag

**Affiliations:** 1 Community Medicine and Primary Care, Umm Al-Qura University, Al-Qunfudah, SAU; 2 Family Medicine, Menoufia University, Shebin Elkom, EGY

**Keywords:** workplace-based, skills, mini-cex, formative assessment, family medicine, dop

## Abstract

Background

Workplace-based assessment (WBA) is a group of assessment approaches that assesses the trainees’ performance through their observation and monitoring in real clinical settings and then provides them with constructive and relevant feedback. Many WBA tools are available, including the mini-clinical evaluation exercise (mini-CEX), direct observation of procedural skills (DOPS), case-based discussions, and multisource feedback (peers, seniors, and patients). A WBA can help medical students improve their clinical competencies and ensure that qualified physicians graduate.

Methods

This prospective study was done in the family medicine department at the Menoufia Faculty of Medicine in Egypt and passed through two phases. Phase I was introducing an orientation lecture for family medicine staff and a convenient sample of 21 family medicine postgraduates about WBA. Phase II was conducting a monthly mini-CEX and DOPS for the postgraduates. Finally, students’ satisfaction with the WBA was assessed, and all collected data were analyzed via Statistical Package for Social Science (SPSS) version 23 (IBM Corp., Armonk, NY).

Results

A total of 105 feedback sheets were obtained. These feedback sheets were subdivided into 63 mini-CEX feedback sheets (21 sheets from each mini-CEX session for three sessions) and 42 DOPS feedback sheets (21 sheets from each DOPS session for two sessions), all of which were collected and analyzed. A significant improvement was detected in the mini-CEX and DOPS feedback scores of the postgraduates throughout the consecutive sessions (9.5 ± 2.7, 24.9 ± 2.5, 27.29 ± 1.5) *(P *< 0.001) for Mini-CEX and (6.1 ± 1.8 versus 9.0 ± 1.2) *(P* < 0.001) for DOPS. About 93% of the postgraduates recommended the application of WBA for their peers, and 86% of them requested to perform it again for other different clinical cases and procedures.

Conclusion

Workplace-based assessment in the form of Mini-CEX and DOPS revealed its ability to improve clinical knowledge and skills among family medicine postgraduates who became motivated to undergo it again in search of improving their clinical performance and reducing their stresses related to final summative and objective structured clinical examinations (OSCEs).

## Introduction

In the health care system, family doctors have a fundamental role to play in achieving health reforms and policy goals. Both access to care and its quality are essential components of health insurance and universal health coverage [1]. Family physicians with additional postgraduate clinical training to become specialists can improve the quality of primary and district-level care when they are part of healthcare teams [2,3] through their roles as clinicians, consultants, capacity builders, clinical trainers, leaders of clinical governance, and supporters of community-oriented primary care [4].

In medical education, evaluating clinical performance is essential but difficult. In the past, evaluations have been implicit, unreliable, and reliant on individual or subjective judgments (the apprenticeship model) [5]. However, recent changes to postgraduate medical education have introduced new methods for evaluating students' performance and competency [6]. One of these systems is a workplace-based assessment, which is an examination of what doctors really do in practice as defined by the assessment of day-to-day procedures conducted in the working environment. Although a doctor's knowledge or competency can be demonstrated in a variety of ways, there is evidence that competency does not consistently predict performance in clinical practice. The capacity to measure performance at the workplace is a significant benefit [7,8].

There are several workplace-based assessment (WBA) techniques that all try to evaluate different aspects of trainee performance. Direct observation of procedural abilities (DOPS) and the mini-clinical evaluation exercise (mini-CEX) are examples of WBA. "Mini-Cex" is an abbreviation for "mini clinical exercise," and it is called "mini" because the trainers conduct it in a short time (about 15 minutes) in comparison to a traditional case evaluation that consumes approximately an hour to be evaluated and discussed with the examiner. During mini-CEX, a healthcare institution's trainee-patient contact is evaluated by an assessor. These clinical interactions are anticipated to take approximately 15 minutes, during which the trainee is expected to complete a focused history, and the physical examination then offers a diagnosis and a treatment strategy for the patient. The performance is then rated using a systematic evaluation form, and helpful criticism is offered [9].

Direct observation of procedural skills (DOPS) was introduced by the Royal College of Physicians and now forms an integral component of the WBA for doctors in the foundation year and those in specialist training. The main goal of DOPS is to evaluate certain procedural skills of medical trainees on real patients in a single encounter by an assessor who then analyzes the trainee's performance, and then a face-to-face feedback session is held [10,11]. In the UK, it has been demonstrated that such an approach to evaluation is valid, reliable, and practicable when evaluating postgraduate medical registrars [12]. For trainees looking to improve their performance in a type of skill, DOPS is regarded as a valuable learning opportunity. Its timely and efficient operation requires detailed collaboration between the assessor and the learner [13]. Therefore, this work was done to detect changes in the clinical performance of family medicine postgraduates after the application of WBA in the form of three consecutive mini-CEX and two successive DOPS as formative assessment, in addition to evaluating the students' satisfaction with such a type of assessment.

## Materials and methods

Study design and setting

A quasi-experimental prospective study was done in the family medicine department at the Menoufia Faculty of Medicine, Egypt. This faculty was established in 1976 and is located in Shebin El-Kom city, in Egypt's Nile Delta and the capital of the Menoufia Governorate.

Participants

This study recruited a convenient sample of 21 postgraduate students who represented all registered postgraduates at the time of data collection.

Procedure

Data were collected over a 12-week study period (from January to March 2020) and passed through two phases. The first phase involved implementing a WBA awareness lecture for family medicine staff and postgraduates. This lecture was introduced over a duration of 120 minutes, and its objectives were to explain the definition of workplace-based assessment, its different approaches, how to conduct it, and the privileges and challenges of its implementation. The second phase involved rolling out a monthly mini-CEX and DOPS for the participating postgraduates for three consecutive months (January, February, and March 2020). Mini-CEX was conducted in a family medicine clinic on a selected case from their curriculum (chronic obstructive pulmonary disease) using a checklist designed according to the Royal College of General Practitioners.

**Figure 1:**
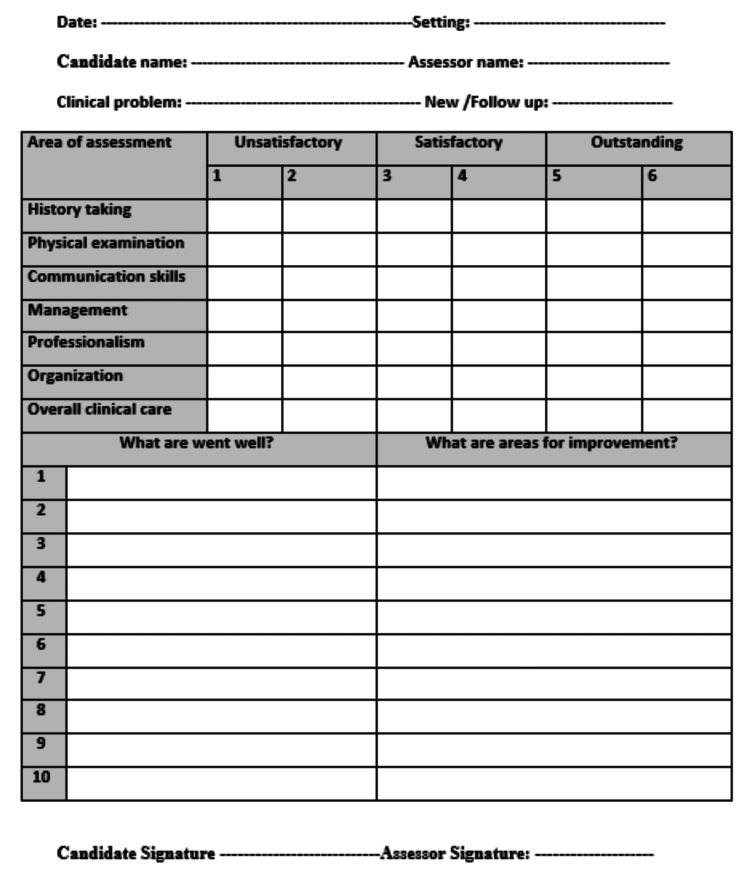
Mini-CEX checklist Mini-CEX: mini-clinical evaluation exercise

Each student was given 15 minutes to conduct his or her mini-CEX, followed by five minutes to receive insightful feedback from his or her observers, who recorded their performance using a predesigned checklist (Figure 1). 

This checklist involved items related to history-taking, physical examination skills, communication skills, clinical judgment, professionalism, organization and efficiency, and overall clinical care (Table 1) [14].

**Table 1:** Specific competencies assessed on mini-CEX Mini-CEX: mini-clinical evaluation exercise

Assessment area	Details of assessment
History-taking	Facilitates the accurate collection of a patient's medical history.
	Effectively applies questions to get the necessary information.
	Creates trust and confidentiality and demonstrates respect, compassion, and empathy.
Physical Examination	Follows an effective, logical sequence balances in problem diagnosis.
	Screening caring about safety.
	Respect for the patient.
Communication Skills	Communicates effectively with patients and their relatives.
	Explains the purpose of the test or treatment and gets the patient's consent.
	Teaches/advises on disease management.
Management	Makes an appropriate diagnosis and management.
	Considers risks and benefits of prescribed treatment.
Professionalism	Demonstrates respectful and professional behavior when interacting with patients, their attendants, and other professionals (e.g., peers, consultants, nursing, professionals, and support personnel).
	Accepts and completes responsibilities with displays ethics.
Organization/Efficiency	Prioritizes; is timely and succinct; summarizes.
Overall Clinical Competence	Demonstrates judgment, synthesis, caring, effectiveness, and efficiency in patient care.

In the mini-CEX, a six-point rating scale was used: unsatisfactory if the score was two or less, satisfactory if the score was three or four, and a performance score of five to six was considered to be above expectations.

**Figure 2:**
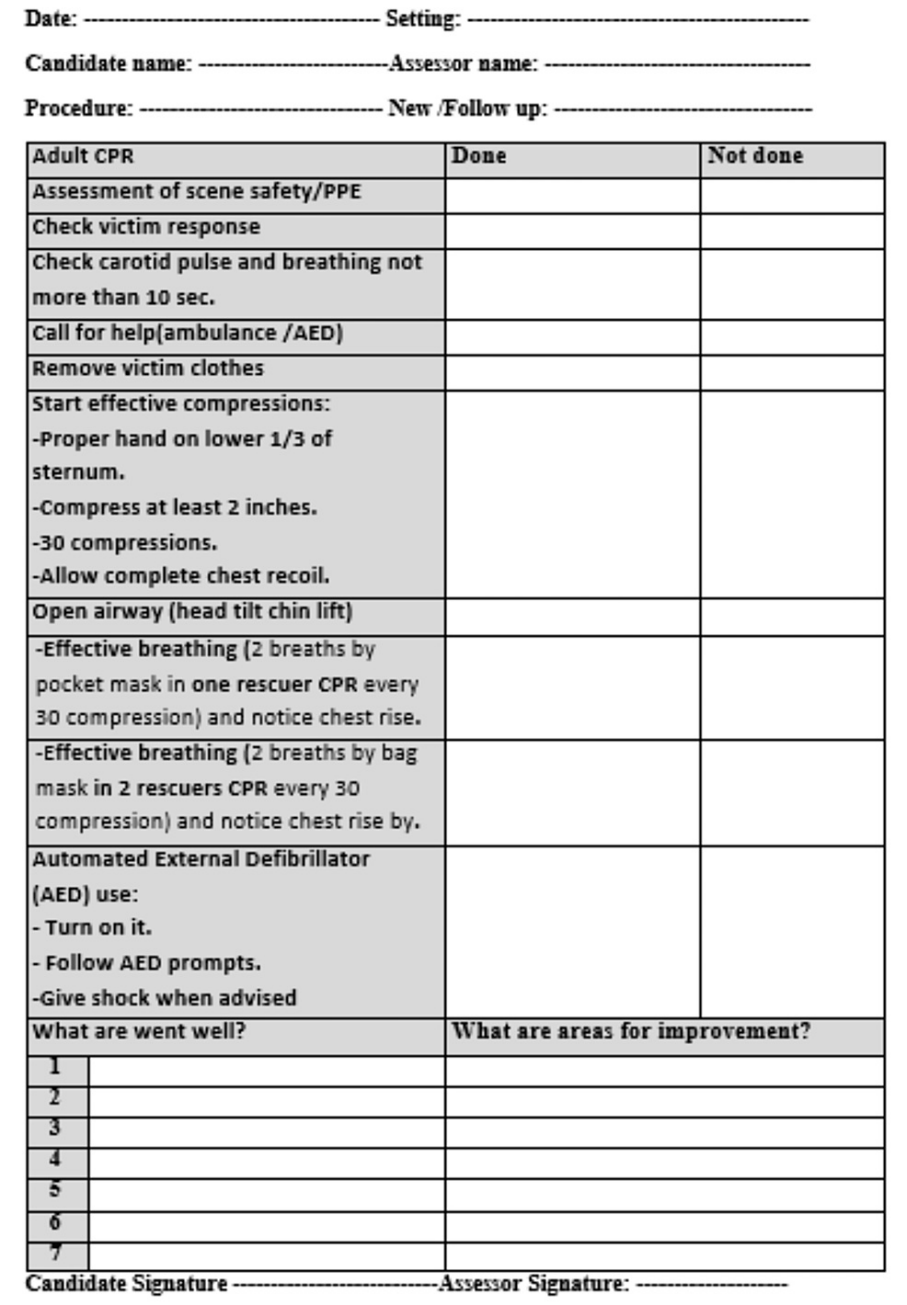
Checklist of direct observation of procedure skills (DOPS) for adult cardiopulmonary resuscitation (CPR) Personal protective equipment (PPE). Cardiopulmonary resuscitation (CPR). Automated external defibrillator (AED).

The postgraduates were subjected to DOPS on adult cardiopulmonary resuscitation (CPR) in the skills laboratory of Menoufia University for two consecutive months and were monitored by their assessors via a checklist prepared according to the American Heart Association and received overall 42 feedback sheets of DOPS on adult cardiopulmonary resuscitation (CPR) that subdivided into 21 feedback sheets from each DOPS session (Figure 2).

Rating Scale

Scoring for the student's performance during DOPS was conducted in the form of a score of 1 when the procedure was done, and a score of 0 if it was not done or incorrectly done.

Each student has received both verbal and written feedback about his or her performance at the end of each session (mini-CEX or DOPS). Finally, an online survey designed on a Google Form composed of 10 questions was used to evaluate their satisfaction with the applied WBA. It involved three questions about mini-CEX (whether it helps them understand the required material, provides them useful feedback on how they performed, and if the assessors gave constructive feedback to students about their mini-CEX performance), and four questions about DOPS (its benefits in understanding the contents of CPR and choking, the provision of effective feedback for each student as regards DOPS performance, and whether constructive feedback about their DOPS performance was given friendly by their assessors). Additionally, the participants were asked about their willingness to take this type of formative exam again and whether they would recommend it to other medical students or not. A five-point Likert scale was used to assess student satisfaction with WBA: strongly agree, neutral, agree, disagree, and strongly disagree.

Ethical considerations

The Institutional Ethical Committee Board of the Faculty of Medicine, Menoufia University, Egypt, approved this study and ensured the confidentiality of data vide IRB 3/2020 FAML 2-C. Verbal and written consent was obtained from each participant after complete disclosure of the research objectives and approach during the awareness lecture. They completely volunteered to participate or not, without any pressure.

Statistical analysis

Data were encoded and entered into Microsoft Excel 2013 (Microsoft Corporation, Redmond, WA) and Statistical Package for Social Science (SPSS) version 23 (using an IBM personal computer). Quantitative data were expressed as means and standard deviations (XSD) and analyzed by a student t-test. An analysis of variance (ANOVA) test was utilized to detect statistical differences among the means of mini-CEX change scores and a paired t-test to determine statistical differences among the means of DOPS scores.

## Results

A total of 21 family medicine postgraduates were involved in this study; their ages ranged from 26 to 32 years, with a mean ±SD of 27.7±1.68 years and a female sex predominance of 19 females (90.9%).

There was a significant improvement in the mini-CEX feedback scores of the postgraduates in the three consecutive sessions regarding medical history, physical examination, and evaluating communication skills, management, organization, and professionalism (9.5 ± 2.7, 24.9 ± 2.5, and 27.29 ± 1.5) (P < 0.001) (Table 2).

**Table 2:** Changes in mini-CEX feedback scores of the family medicine postgraduates F = One-way ANOVA test. Statistically significant (P value <0.05) Mini-CEX: mini-clinical evaluation exercise; ANOVA: analysis of variance

Items of assessment	First mini-CEX	Second mini-CEX	Third mini-CEX	F	P value
History taking	1.19± 0.40	3.42± 0.67	4.00±0.55	151.14	<0.001
Physical examination	1.19± 0.40	3.38 ± 0.74	3.60±0.49	117.96	<0.001
Communication skills	2.00± 1.34	4.04±0.59	4.10±0.0.57	37.29	<0.001
Management	1.61±0.92	3.48±0.51	3.90±0.36	72.96	<0.001
Professionalism	1.19± 0.60	3.57±0.51	3.90±0.54	151.78	<0.001
Organization	1.09±0.30	3.57±0.59	4.00± 0.63	182.75	<0.001
Overall clinical care	1.19±0.51	3.38± 0.67	4.00±0.63	110.30	<0.001
Total score	9.5 ± 2.70	24.9 ± 2.50	27.29 ± 1.50	379.00	<0.001

There was a significant improvement in the DOPS feedback scores of the participants for two consecutive months in the skills laboratory (6.1 ± 1.8 versus 9.0 ± 1.2) (P < 0.001) (Table 3).

**Table 3:** Changes in feedback scores of direct observation of procedures (DOPS) of adult CPR of the family medicine postgraduates PPE: personal protective equipment; CPR: cardiopulmonary resuscitation Statistically significant (P value<0.05)

Items of assessment	First DOPS	Second DOPS	Paired T-test	P value
-Assessment of scene safety/PPE	0.62±0.49	0.86±0.36	2.02	0.060
-Check victim response	0.52±0.51	1.00±0.00	4.26	<0.001
-Check carotid pulse and breathing not more than 10 seconds	0.67±0.48	1.00±0.00	3.16	0.005
-Call for help (ambulance/AED)	0.48±0.51	0.81±0.40	2.32	0.031
-Remove the victim's clothes	0.71±0.46	0.90±0.30	1.45	0.162
-Start effective compressions: Proper hand placement at the lower 1/3 of the sternum. Compress at least 2 inches. Conduct 30 compressions. Allow complete chest recoil	0.62±0.49	0.95±0.22	2.65	0.021
-Open airway (head tilt chin lift)	0.67±0.48	1.00±0.00	3.16	0.005
-Effective breathing (2 breaths every 30 compressions) notice chest rise by pocket mask in one rescuer CPR	0.81±0.40	0.86±0.36	0.44	0.671
-Effective breathing (2 breaths every 30 compressions) notice chest rise by bag-mask in 2 rescuers CPR	0.52±0.51	0.76±0.43	2.02	0.060
-Automated External Defibrillator (AED) use: Turn on it. Follow AED prompts. Give shock when advised	0.48±0.51	0.86±0.36	2.96	0.008
-Total DOPS score	6.09±1.84	9.00±1.22	5.79	<0.001

**Figure 3:**
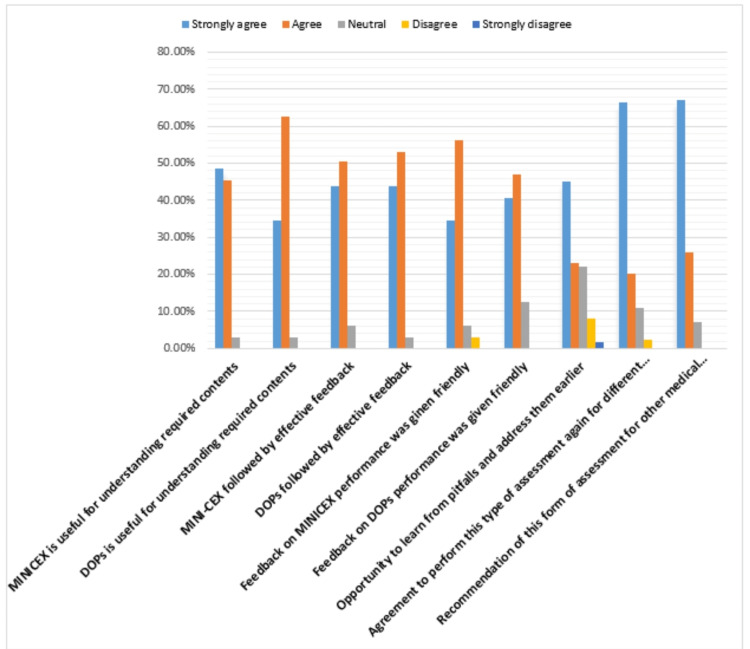
Satisfaction of the family medicine postgraduates with WBA WBA: workplace-based assessment

About 93% of participating postgraduates recommended the application of WBA for other medical students, and 86% of them were motivated to perform it again for other different clinical cases and procedures (Figure 3).

## Discussion

Medical school graduates will face a variety of healthcare scenarios, all of which require professional, competent, and skillful responses. This is because the field of medicine is highly complex [15]. During training, evaluation plays a significant role in helping the trainees identify their strengths and areas for improvement. It also assists them in developing the skills necessary to handle a variety of situations while considering the patient's safety. Medical graduates who participate in WBA have a unique opportunity to advance professionally based on feedback from workplace-based evaluation assessors [16].

The goal of this study was to conduct an awareness lecture about WBA for family medicine postgraduates and staff members, then administer three mini-CEX and two DOPS to the postgraduates and detect changes in their performance over a three-month period while being monitored by the assessors.

The mini-CEX feedback scores of the postgraduates for medical history, physical examination, and evaluating communication skills, management, organization, and professionalism showed a significant improvement in all clinical care scores over three consecutive sessions (9.5±2.7, 24.9±2.5, and 27.29±1.5) (P = 0.001). Many previous researchers found that the mini-CEX positively affects the learning process [17,18]. Students will spend more time practicing history taking and physical examinations if they perform mini-CEX on a regular basis. Mini-CEX provides a positive experience, both in terms of knowledge and clinical skills [19]. This finding coincides with that of Vaughan et al. (2017), who studied students' learning response toward feedback during mini-CEX on 24 participants (9 males and 15 females) while they were undergoing clerkship in the internal medicine rotation in order to prevent recall bias [20]. Feedback content is useful to describe the student’s performance in the achievement of competence and the performance gap, and it is important to improve the students’ learning response. Khalil et al. (2017) assessed clinical competencies in pediatrics in 20 postgraduate students (12 males and 8 females) using mini-CEX; they found that feedback has a positive impact on student's future performance [21]. This could be one of the main advantages of involving mini-CEX in the formative assessment of postgraduates.

There was a significant improvement in all DOPS feedback scores of the family medicine postgraduates (6.1 ± 1.8 versus 9.0 ± 1.2) (P < 0.001). This is consistent with numerous results from studies by Hengameh et al. (2015) and Roghieh et al. (2013), which compared the effects of DOPS with the standard evaluation method on Iranian nursing students. The two studies revealed that DOPS significantly increased clinical skills (P = 0.000) [22,23]. Additionally, Shahgheibi et al. (2009) evaluated the effects of DOPS on students' learning levels during clinical externships in the obstetrics department. The results of another study conducted in Iran with 73 medical students (42 control and 31 intervention) showed that DOPS could be more effective at enhancing students' skills than the control group (P = 0.001) [24].

Furthermore, Profanter and Perathone (2015) conducted a prospective randomized experiment in Austria on 193 students in a surgical skills lab course; the DOPS group demonstrated a higher degree of clinical skills than the control group, and DOPS dimensions appeared to enhance tutoring and performance rates [25]. In 2014, in India, Dabhadkar et al. examined how DOPS has affected second-year postgraduate obstetrics and gynecology students' learning and found that five out of six students who had unsatisfactory performance in the first round of DOPS improved to a satisfactory level in the second round [26]. Bagheri et al. (2014) examined the effects of DOPS on postgraduate students' learning in Iran studying emergency medicine (25 in the experiment and 21 in the control group). In comparison to the control group, the experimental group's mean scores were significantly higher (P = 0.0001, t = 4.9) [27]. All of the previously mentioned data reflect the important role of DOPS in improving students' performance. Indeed, close monitoring and positive, constructive feedback are the root causes of such improvement.

In this study, about 93% of students recommended the application of workplace-based assessment for other medical students, and 86% of them agreed to perform it again for other clinical cases and procedures. It is consistent with the findings of a 2017 study of surgical residents at an Indian government medical college and tertiary care teaching hospital who readily accepted the WBA tool. Their great level of satisfaction with the performance provided as evidence of their ability to continuously improve in the identified weak areas is supported by an improvement in residents' performance over the WBA period [28]. As per the Tenzin et al., 2019 study, obstetrics and gynecology residents were the most satisfied with workplace assessments at 90%, followed by pediatricians at 80% [29].

This study had some limitations that should be enumerated. First, after recruiting the study sample and collecting data in January 2020, the coronavirus disease 2019 (COVID-19) pandemic spread throughout the world, so we suspended data analysis and writing this article until the end of 2021 because the state of emergency was lifted at all hospitals, and we were assigned, like all medical teams, to confront the coronavirus pandemic in addition to our work as medical educators and the sudden shift to virtual learning in our medical school. Second, there was grader bias, as only two observers evaluated the postgraduates due to the decreased number of staff members who attended the awareness lecture and were willing to participate in the study but, due to their work overloads, they could not. However, this study highlighted the importance of two forms of WBA for promoting students' clinical competencies. Actually, it was a very interesting experience for both the investigators and the candidates. Finally, after discussing the study findings with our department's family medicine staff, they decided to implement them as frequently as possible for all postgraduates.

## Conclusions

Based on the findings of this study, we can conclude that WBA in the form of mini-CEX and DOPS improves the clinical competencies of family medicine postgraduates and increases their acceptability through the use of this type of formative assessment and its repetition for future cases and procedures. Therefore, this study emphasizes the need for more endorsing WBA applications for all medical specialties, not only in our faculty of medicine but also in all medical colleges worldwide. Further, similar studies are recommended to support the data derived from the current study, notably this study, which was conducted in only one context and investigated only one competency, whether in the mini-CEX or DOPS.
